# Polycarbonate Composites Reinforced with *Spartium junceum* L. Fibers: A Promising Material for the Automotive Industry

**DOI:** 10.1021/acsomega.6c02449

**Published:** 2026-07-10

**Authors:** Mario Meheš, Emi Govorčin Bajsić, Dražan Jozić, Sanja Perinović Jozić

**Affiliations:** † University of Zagreb Faculty of Chemical Engineering and Technology, Trg Marka Marulića 19, Zagreb 10000, Croatia; ‡ Faculty of Chemistry and Technology, University of Split, Ruđera Boškovića 35, Split 21000, Croatia

## Abstract

The growing demand for sustainable materials has intensified research into natural fiber-reinforced polymer composites as alternatives to conventional fossil-based plastics. In this study, polycarbonate (PC) composites reinforced with Spartium junceum L. (SJL) fibers were developed and systematically characterized to evaluate their potential for automotive applications. Composites containing 5–20 wt % of randomly oriented short SJL fibers were prepared and analyzed in terms of thermal, mechanical, and morphological properties. Dynamic mechanical analysis revealed increased stiffness with rising fiber content, accompanied by a slight reduction in glass transition temperature. Tensile testing showed significant improvements in strength and Young’s modulus, with optimal performance observed at 10–15 wt % fiber loading, attributed to improved fiber dispersion and interfacial adhesion. At higher fiber content (20 wt %), mechanical performance declined due to fiber agglomeration and reduced stress transfer efficiency. Thermal analysis indicated good compatibility between SJL fibers and the PC matrix, although a gradual decrease in thermal stability was observed with increasing fiber content. Scanning electron microscopy confirmed generally adequate fiber–matrix interaction, particularly at intermediate fiber loadings. Swelling tests demonstrated minimal water uptake, indicating preserved hydrophobicity of the composites. Overall, the results highlight the potential of SJL fibers as an effective, sustainable reinforcement for polycarbonate composites, offering improved mechanical performance while contributing to environmentally friendly material solutions for the automotive industry.

## Graphical abstract

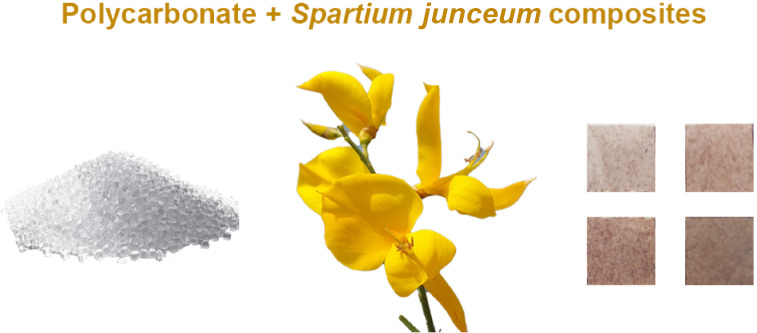

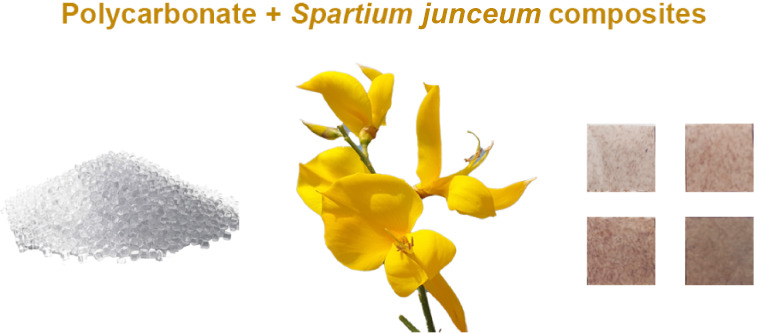

## Introduction

Natural fibers have received growing attention in recent decades due to their renewability, biodegradability, low environmental impact, and strong mechanical properties. These attributes drive their use across industries, fostering sustainable innovation. Natural fibers are now used across a wide range of industries: textiles remain their traditional and most widespread application, while numerous review studies confirm their increasing integration into automotive composites, construction materials, paper and pulp, sustainable packaging, aerospace components, and sports equipment.^1–5^ Natural fiber-reinforced polymer composites offer compelling benefits for automotive applications: lightweight: 15–25% mass reduction vs glass fiber composites, boosting fuel efficiency by 5–10% and EV range;^6^ mechanical performance: tensile modulus ∼20–30 GPa with PLA matrix, rivaling E-glass in nonstructural parts like door panels (improved 20% vibration damping from lignin);^7^ sustainability: local sourcing cuts transport emissions 40–60%; fully biodegradable end-of-life vs landfilled synthetics; aligns with EU Green Deal 55% CO_2_ reduction target by 2030;^6,8^ cost/ease: 20–30% cheaper raw materials/processing than carbon fiber; simple alkali treatments enhance fiber-matrix bonding.^9^

*Spartium junceum* L. (SJL) also known as Spanish Broom,^10,11^ is a perennial flowering shrub in the legume family that is highly characteristic of Mediterranean landscapes due to its bright, fragrant yellow blossoms. This resilient and drought-tolerant plant is well adapted to sunny, arid environments, where it is frequently used to help prevent soil erosion. Historically, its long, flexible stems were harvested to produce fiber for rope and brushes, while today it is widely cultivated as an ornamental shrub for its aesthetic and ecological value.^12^ SJL consists of cellulose (60–70%), hemicellulose (15–20%), lignin (10–15%), and waxes/pectins (<5%).^13,14^ Exact component ratio depends on extraction method.^13,15^ Mechanical properties of SJL fibers are comparable to other natural fibers (Table 1) actively used by many manufacturers in various components of modern vehicles.^16–20^ Given pure fiber properties, plant resilience, and availability in Mediterranean region, SJL has good potential to be used in polymer composites.

**Table 1:** Mechanical Properties of Natural fibers^7,21^

Fiber Type	Tensile Strength, MPa	Young’s Modulus, GPa	Elongation at Break, %
Flax (*Linum usitatissimum*)^a^	287–800	5–13	3–10
Hemp (*Cannabis sativa*)^a^	550–900	70	1–6
Jute (*Corchorus* *spp.*)^a^	390–800	10–55	1–2
Kenaf (*Hibiscus cannabinus*)^b^	295–930	53	2–7
Sisal (*Agave sisalana*)^a^	511–635	9–28	2–3
Spanish Broom (*Spartium junceum*)^b^	914	17	6

a Data adapted with permission from ref. 21. Copyright 2023 The Authors. Published by Sveučilište u Zagrebu Tekstilno-tehnološki fakultet.

b Data adapted from ref. 7 under the terms of the Creative Commons CC BY license. Copyright 2025 The Authors. Published by TANGER Ltd.

This paper systematically synthesizes the results of our previous findings^22,23^ to assess overall trends that were not apparent in individual papers. This synthesis is essential to the interpretation of the new results and the integrity of the scientific argument.

## Experimental Section

In this study, polycarbonate (PC) Makrolon 2805, in pellet form, was supplied by Bayer AG (Germany). According to the manufacturer’s specifications, the material exhibits a melt flow index (MFI) of 10 g/10 min at 300 °C, indicating its suitability for thermoplastic processing techniques such as injection molding and extrusion. *Spartium junceum* L. fibers were sourced from wild-growing plants harvested in the vicinity of Šibenik, Croatia. Prior to characterization and composite fabrication, the fibers were extracted from the plant material through a maceration process as reported by Kovačević^24^ Exact chemical composition of the used SJL fibers is reported in earlier work in accordance with the TAPPI T 211 om-02 ^25^ (0.03% ash), TAPPI T 204 cm-97 ^26^ (1.7% extractive content), TAPPI T 222 om-11 ^27^ (3.5% lignin), and Küschner–Hoffer method (91% cellulose, 3% hemicellulose).

### Preparation of Fibers

Fresh stems of *Spartium junceum* L. were immersed in 5% (w/v) NaOH solution (the ratio of stems to solution was 1:12 g/mL). After 5 min running at 900 W in the microwave, stems were washed in distilled water, and the fibers were pulled out by hand. The fibers were washed again in distilled water until a neutral pH was reached. After washing, the fibers were air-dried. The dried fibers were cut with scissors to a length shorter than 7.5 mm so they can be classified as short fibers per ISO 10350–1.^28^

### Preparation of Composites

To investigate the influence of *Spartium junceum* L. (SJL) fibers on the properties of a polycarbonate (PC) matrix, a series of composite materials was prepared with varying fiber contents (5, 10, 15, and 20 wt %) using randomly oriented short fibers. The composites were compounded in a Brabender GNF106/2 mixer at 190 °C, with a screw speed of 60 rpm, for 5 min (2 min PC + 3 min SJL). The compounded materials were then molded using a Fontijne SRB 140 hydraulic hot press (Netherlands) at 190 °C under a pressure of 11 MPa for 5 min in 100 × 100 × 1 mm mold with 5 min of preheating. The molds were cooled with water to room temperature inside the press to ensure uniform solidification. Cooling speed was approximately 3.5 °C/min. The composite preparation procedure is illustrated in Figure 1.

**Figure 1:**
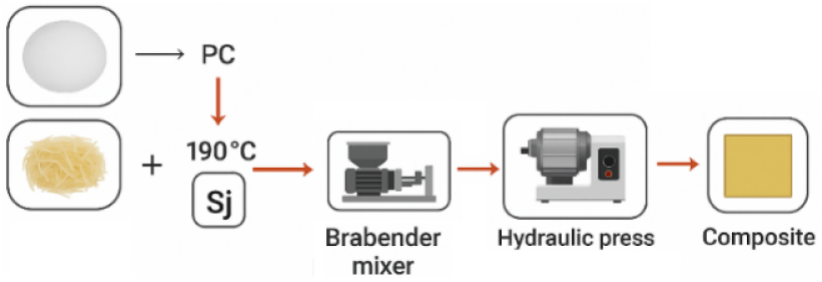
Preparation of composites.

### Differential Scanning Calorimetry (DSC)

The thermal properties of the composites were analyzed using differential scanning calorimetry on a Mettler Toledo DSC 822e instrument (Switzerland), operated under a continuous flow of dry nitrogen gas (40 mL·min^–1^). Samples were cut with a utility knife to 10.25 ± 0.25 mg. Each sample was heated to 190 °C and held at that temperature for 5 min. The samples were then cooled to −100 °C at a rate of 10 °C·min^–1^ (cooling cycle), held at −100 °C for 5 min, and subsequently reheated to 190 °C at the same rate (heating cycle). All measurements were performed under a nitrogen atmosphere (flow: 40 mL/min, purity: 99.9990%) to prevent oxidative degradation. Samples were measured in 40 μL aluminum crucibles with pierced lid.

### Thermogravimetric Analysis (TGA)

The thermal stability of the composites was assessed using thermogravimetric analysis on a TA Instruments Q500 (USA). Samples were cut with a utility knife to approximately 10 ± 0.5 mg. Each sample was heated from room temperature to 600 °C at a constant heating rate of 10 °C·min^–1^ under a nitrogen flow of 60 mL·min^–1^ (purity: 99.9990%). The temperature corresponding to a 5% weight loss (T_5%_) and the temperature at the maximum decomposition rate (*T*_max_) were determined from the resulting TGA curves.

### Dynamic Mechanical Analysis (DMA)

Dynamic mechanical analysis was performed using a TA Instruments DMA 983 to evaluate the viscoelastic properties of the composites. The measurements were carried out over a temperature range from room temperature to 200 °C, at a fixed frequency of 1 Hz and an oscillation amplitude of 0.3 mm. The heating rate was maintained at 3 °C·min^–1^. Samples were cut with a utility knife to an approximate width of 10 mm. The length of the sample was 25 mm. The clamp used was a serrated vertical clamp.

### Mechanical Properties

The tensile properties of the composites were determined using a Zwick Roell Z020 universal testing machine (Germany) at a crosshead speed of 50 mm·min^–1^. Samples were cut with a utility knife into strips with an approximate width of 10 mm. The length of the sample was 100 mm, and the distance between clamps was set to 50 mm. To ensure statistical reliability, a minimum of five specimens were tested for each composition.

### Fourier Transform Infrared Spectroscopy (FTIR)

The surface chemical properties were analyzed by Fourier Transform Infrared Spectroscopy using a PerkinElmer Spectrum 100 spectrometer (USA) in attenuated total reflectance (ATR) mode. Spectra were recorded at room temperature under ambient humidity, over a wavenumber range of 4000–650 cm^–1^, with a resolution of 4 cm^–1^. Samples were analyzed as is without any preparation.

### Scanning Electron Microscopy (SEM)

Morphological analysis of the PC, SJL, and PC/SJL composites was conducted using scanning electron microscopy with a JEOL JSM-7610F (Japan) microscope operated at an accelerating voltage of 1.00 kV. Samples without conductive coating were examined at various magnifications to assess fiber dispersion and interfacial adhesion within the matrix.

### Swelling Test

The swelling test was conducted following ISO 62 Method 1 ^29^ on samples of neat PC and PC/SJL composites, with three specimens prepared for each composition. Specimens measuring 20 × 20 × 1 mm were cut with a utility knife, and their initial mass was determined using an analytical balance. The samples were then fully immersed in beakers containing distilled water at room temperature (24 °C). After 1, 3, 6, 14, 21, and 28 days, the samples were removed from the water, carefully dried with a lint-free cloth, and promptly weighed with an accuracy of 0.001 g to determine their mass after swelling. The change in mass was used to calculate water uptake. The percentage of weight absorption was calculated using the following and plotted as a function of water exposure time.

$$w_{\mathrm{ab}}=\frac{w_{\mathrm{tm}}-w_{0}}{w_{0}}\times 100$$

where ω_0_ is the weight of the sample before and ω_tm_ after being exposed to water.

### X-ray Diffraction Analysis (XRD) and X-ray Microcomputed Tomography (XμCT)

X-ray diffraction analysis was performed to determine the crystalline structure and phase composition of the composites. Analysis was performed on samples using Malvern PANalytical Empyrean X-ray diffraction system (UK). The system used a copper X-ray tube (λKα1 = 1.5406 Å), iCore/dCore multicore optics, and a PIXcel3D detector. The diffraction patterns were recorded under the following conditions: 45 kV anode voltage, 40 mA anode current, a step size of 0.013°, and a 2θ scan in the range of 4° to 80°. For qualitative analysis, the XRD patterns were compared with standard patterns from the ICDD database PDF-2 (PDF-2 RELEASE 2024) using Malvern PANalytical HighScore Plus software (UK). XμCT is performed at the same instrument by using the same X-ray tube with a point-focused X-ray beam and CT module. XμCT measurement was performed by using voltage of 15 kV and a current of 10 mA. The object distance from the X-ray source was 452 mm, and the object-detector distance was 28 mm (XμCT geometry). Radiographs are collected withan exposure time of 10 s for each angle of the sample rotation (rate of rotation: 0.4 ° s^–1^). For data collection , the same PIXcel3D detector was used with Medipix3. The window size of the 0D detector was 14 × 14 mm (256 × 256 pixels) with a pixel size of 55 μm and a spatial resolution of 55 × 55 μm. CT reconstruction was performed using VGSTUDIO MAX (Volume Graphics GMBH, Germany). The presence of inclusions in the structure of PC/SJL 15 composite was analyzed using Porosity/Inclusion Analysis module.

## Results and Discussion

### Viscoelastic Properties

In the context of natural fiber-reinforced biocomposites, the dynamic mechanical properties are influenced by several key factors: the intrinsic properties of the polymer matrix, the nature and morphology of the reinforcing fibers, and, most importantly, the quality of interfacial adhesion between the fiber and the matrix. Furthermore, the dispersion and orientation of the fibers within the matrix play a significant role in determining the overall mechanical performance under dynamic stress. Good interfacial bonding provides effective stress transfer from the matrix to the fibers, thus enhancing the stiffness and damping capacity of the composite. Conversely, poor interaction or agglomeration of fibers may result in stress concentration and diminished mechanical performance. Figure 2a shows the variation of the storage modulus (E′) as a function of temperature for neat PC and PC/SJL composites reinforced with different weight contents of SJL fibers. In the glassy region, i.e., at temperatures below the glass transition temperature (*T*_g_) all samples exhibit a relatively stable storage modulus, with no significant deviations observed between the neat PC and the fiber-reinforced composites. This plateau behavior indicates a rigid and stiff material phase where molecular mobility is highly restricted. As the temperature increases beyond *T*_g_, the materials transition into the rubbery plateau region, characterized by a decrease in the storage modulus due to increased segmental motion of polymer chains. In this region, the storage modulus of the PC/SJL fiber composites demonstrates a notable enhancement with increasing SJL fiber content, indicating that the reinforcing effect of the SJL fibers becomes more pronounced at elevated temperatures. However, an exception is observed in the composite containing 20 wt % SJL fibers, which does not follow the expected trend and shows a lower *T*_g_ and storage modulus compared to composites with lower fiber content (Table 2). At 20 wt %, the nonlinear increase in *T*_g_ and modulus is explained by competing effects of local chain confinement in fiber-rich regions and fiber agglomeration, which introduces interfacial heterogeneity and reduces load transfer efficiency. Overall behavior reflects a balance between reinforcement and structural discontinuity. Observed increases in E’ upon cellulose fiber incorporation are consistent with reinforcement from a percolating cellulose network, which is supported by cellulose’s native semicrystalline structure.^30–32^ The presence of ordered crystalline domains enhances stiffness by restricting polymer chain mobility, thereby improving the composite’s elastic response. At high fiber contents (20 wt %), mechanical performance exhibits a decline. Increasing fiber content beyond the optimum can reduce reinforcement efficiency because fiber agglomeration, voids, and poor interfacial adhesion limit stress transfer and promote crack initiation. Figure 2b shows the temperature-dependent loss modulus (E″) of neat PC and PC/SJL composites. In the case of neat PC, the E″ curve exhibits a broad relaxation peak around 55 °C, corresponding to the so-called β-relaxation. This secondary relaxation phenomenon is typically associated with the local motion of short polymer chain segments or side groups within the amorphous regions of the polymer.^33^ Such relaxations do not involve the cooperative motion of the entire polymer backbone but are indicative of increased segmental mobility at subglass transition temperatures. At higher temperatures, a well-defined, intense peak at approximately 145.7 °C is observed, representing the glass transition temperature (*T*_g_) of PC. For the PC/SJL fiber composites with increasing SJL fiber content, a progressive shift of the *T*_g_ peak to lower temperatures is evident (Table 2). This decrease in *T*_g_ suggests an enhancement in chain mobility within the amorphous phase of PC, which may be attributed to the presence of lignin in the SJL fibers. Lignin, an amorphous and hydrophobic biopolymer, can act as a plasticizer or disrupt polymer interactions, reducing the rigidity of the PC matrix. Similar trends were observed in nanocellulose/PC composites^34^ where natural fibers or lignocellulosic fillers reduced *T*_g_, likely due to interfacial changes and increased free volume. These findings highlight the combined effect of fiber composition and polymer structure on the thermomechanical behavior of biocomposites.

**Figure 2:**
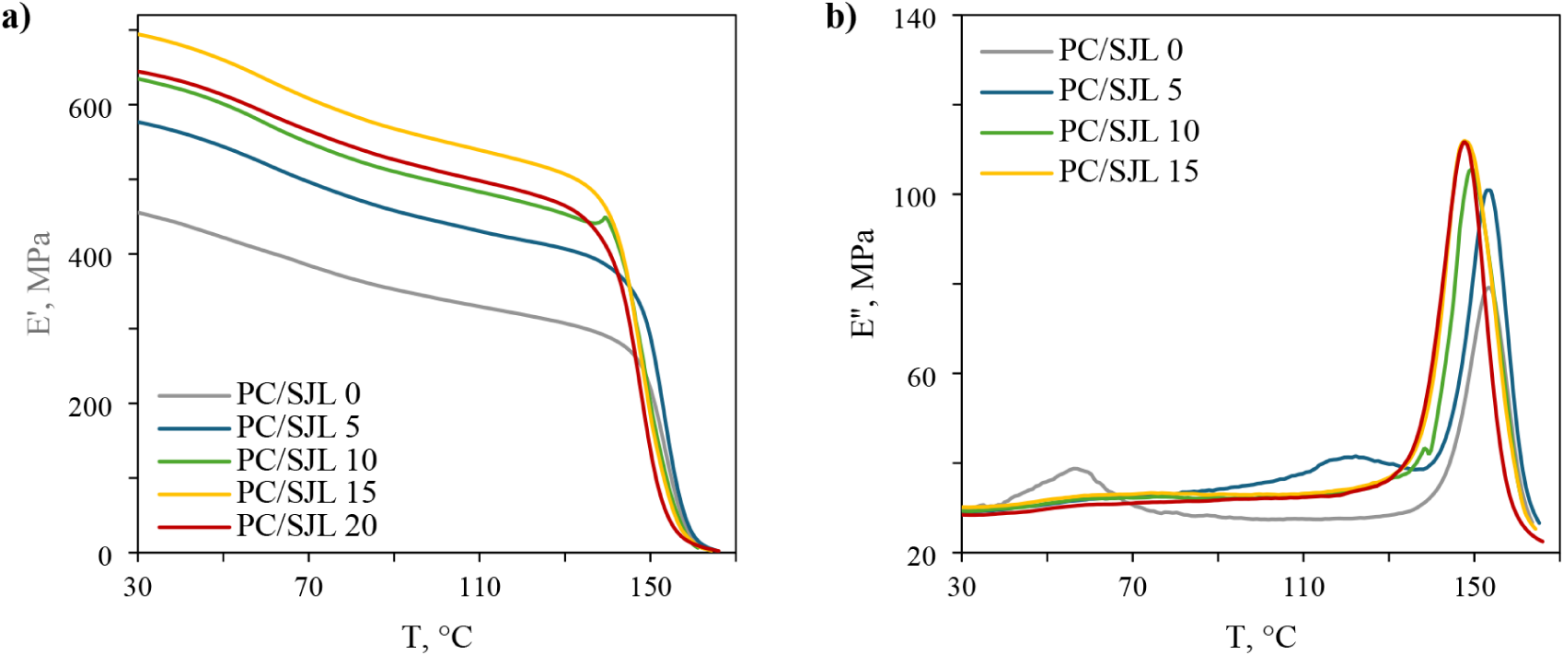
(a) Storage modulus (E’) and (b) loss modulus (E’’) as a function of temperature for neat PC and PC/SJL fiber composites. Adapted with permission from ref. 22 under the terms of the Creative Commons CC BY license. Copyright 2025 The Authors. Published by TANGER Ltd.

**Table 2:** DMA Analysis Results for PC and PC/SJL Composites^a^

Sample	T_g_, **°**C	E’_25_, MPa
PC/SJL 0	145.7	461.6
PC/SJL 5%	142.4	580.9
PC/SJL 10%	140.6	634.7
PC/SJL 15%	138.8	698.6
PC/SJL 20%	140.8	646.0

a Data adapted from ref. 22 under the terms of the Creative Commons CC BY license. Copyright 2025 The Authors. Published by TANGER Ltd.

### Mechanical Properties

The stress–strain curves of neat PC and composites reinforced with varying contents of SJL fibers are presented in Figure 3. The corresponding values of tensile strength (σ), elongation at break (ε) and Young’s modulus (E) for neat PC and PC/SJL fiber composites are shown in Table 3.

**Figure 3:**
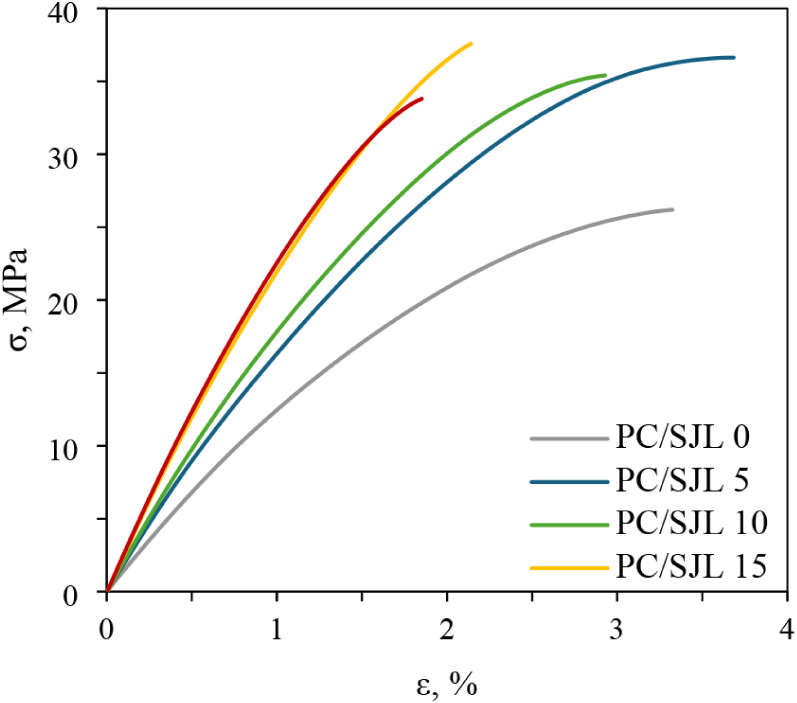
Stress/strain curves for the neat PC and PC/SJL fiber composites. Adapted with permission from ref. 22 under the terms of the Creative Commons CC BY license. Copyright 2025 The Authors. Published by TANGER Ltd.

**Table 3:** Mechanical Properties of Neat PC and PC/SJL Composites^a^

Sample	σ, MPa	ε,%	E, MPa
PC/SJL 0	25.2 ± 0.4	3.7 ± 1.3	13.9 ± 0.3
PC/SJL 5	36.4 ± 0.2	3.1 ± 0.1	18.8 ± 0.4
PC/SJL 10	36.9 ± 3.3	2.9 ± 0.2	21.2 ± 0.3
PC/SJL 15	39.2 ± 5.2	2.1 ± 0.6	26.6 ± 0.4
PC/SJL 20	33.1 ± 8.1	1.8 ± 0.6	25.5 ± 0.1

a Data adapted from ref. 22 under the terms of the Creative Commons CC BY license. Copyright 2025 The Authors. Published by TANGER Ltd.

From the obtained data in Table 3, it can be observed that the addition of SJL fibers up to 15 wt % leads to a significant increase in tensile strength compared to neat PC. The improvement in tensile strength and stiffness at intermediate fiber loadings (10–15 wt %) is attributed to an optimized balance between fiber dispersion, interfacial adhesion, and effective stress transfer across the fiber–matrix interface. Under these conditions, SJL fibers are more uniformly distributed within the polycarbonate matrix and act as effective reinforcements. SEM observations support this interpretation, revealing partial fiber pull-out and fiber fracture, which indicate efficient load transfer prior to failure. In contrast, at higher fiber content (20 wt %), the reinforcement efficiency is significantly reduced due to fiber agglomeration, increased void content, and interfacial debonding. These morphological features disrupt matrix continuity, introduce local stress concentration zones, and hinder uniform stress distribution, leading to premature failure. This reduction at higher fiber contents may be attributed to fiber agglomeration, which creates stress concentration sites and reduces the homogeneity of the composite.^35^ Additionally, the higher fiber content results in a diminished reinforcing effect, likely due to poor dispersion and matrix–fiber incompatibility. These findings are consistent with previous studies on natural fiber–reinforced polymer composites. For example, Bajpai et al.^36^ investigated PLA composites reinforced with natural fibers and reported a similar trend: an increase in tensile strength at lower fiber contents, followed by a decline at higher contents, attributed to insufficient dispersion and increased void content. The observed decrease in tensile strength at higher fiber contents may also be attributed to the inherent hydrophobicity of the polymer matrix and the hydrophilic nature of natural fibers, which together limit effective interfacial bonding. Conversely, improvement in tensile strength at lower fiber contents can be attributed to enhanced interfacial interactions between SJL fibers and the PC matrix, as confirmed by SEM analysis. Morphological observations revealed good fiber–matrix adhesion and uniform fiber distribution in samples containing ≤ 15 wt % fiber content. The fiber–matrix interface plays a critical role in determining the mechanical behavior of fiber-reinforced composites, particularly regarding stress transfer efficiency and failure mechanisms. On the other hand, the elongation at break decreased significantly with increasing fiber content (Table 3), as expected, due to increased stiffness and reduced ductility. The Young’s modulus of the composites increased markedly with the addition of SJL fibers, indicating improved material rigidity. The increases in tensile strength and Young’s modulus are consistent with the rise in storage modulus observed in DMA measurements. Overall, these results demonstrate that SJL fibers are a viable reinforcement for PC, particularly in terms of enhancing strength and stiffness, with optimal performance observed at 10–15 wt % fiber content. Compared to previous research on natural fiber-reinforced PC composites, SJL fibers demonstrate a greater improvement in tensile strength and Young’s modulus (Table 4).

**Table 4:** Comparison of Changes in Mechanical Properties of PC/Natural Fibers Composites

Fiber type	Fiber content (wt %)	Tensile strength change (%)	Young’s modulus change (%)
Pineapple leaf (untreated)^37^	5–20	–8 to −5	–13 to 2
Pineapple leaf (NaOH treated)^37^	5–20	0 to 5	1 to 75
Pineapple leaf (NaOH/silane)^37^	5–20	–11 to 4	–13 to 54
Hemp (NaOH treated)^38^	10–30	–29 to −1	Not reported
Wood (cellulose nanofibril film)^35^	10–32	10 to 31	–50 to 100
*Spartium junceum* L. (untreated)	5–20	30 to 55	35 to 91

### DSC Results

The thermal behavior of PC and PC reinforced with SJL fibers was examined using differential scanning calorimetry (DSC). The glass transition temperature (*T*_g_), melting temperature (*T*_m_), and crystallization temperature (*T*_c_) were determined from the second heating and cooling cycles. The summarized values are presented in Table 5. The DSC thermogram of SJL fibers (Figure 4) exhibited a broad *T*_g_ at approximately 96 °C, attributed to the amorphous nature of lignin in SJL. No distinct melting or crystallization peaks were observed, confirming the predominantly amorphous structure of the fibers.

**Table 5:** DSC Results of Neat PC and PC/SJL Composites^a^

Sample	T_g_, °C	T_m_, °C	T_c1_, °C	T_c2_, °C
PC/SJL 0	146	164	118	100
PC/SJL 5%	142	161	-	99
PC/SJL 10%	141	160	110	97
PC/SJL 15%	139	160	108	98
PC/SJL 20%	141	160	112	109

a Data adapted from ref. 23 under the terms of the Creative Commons CC BY license. Copyright 2024 The Authors. Published by TANGER Ltd.

**Figure 4:**
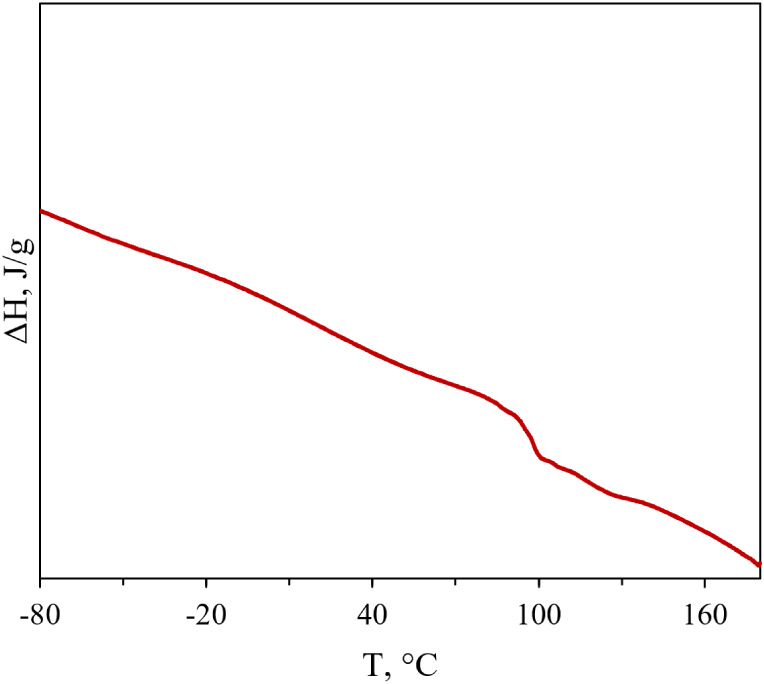
DSC curve of the second heating cycle of SJL.

Figure 5 presents the DSC curve of PC granules and PC processed via melt mixing using a Brabender mixer. Generally, PC is known as a fully amorphous thermoplastic polymer, and its thermal behavior is typically characterized by a distinct *T*_g_. For pristine PC granules (Figure 5a), a *T*_g_ value of 142.5 °C was observed, which is consistent with literature data for commercial-grade PC. The *T*_g_ corresponds to the onset of significant segmental motion of polymer chains and is strongly dependent on the flexibility and mobility of the molecular backbone. In general, greater chain flexibility leads to lower *T*_g_ values, whereas increased rigidity (e.g., due to bulky side groups or chain interactions) results in higher *T*_g_ values.

**Figure 5:**
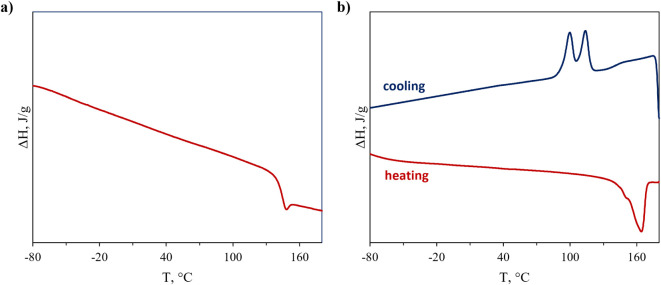
DSC curves of the second heating/cooling cycle of a) PC granules and b) PC processed via melt mixing using a Brabender mixer.

Interestingly, DSC analysis of PC processed via Brabender internal mixing revealed a shift in the *T*_g_ to approximately 145.7 °C. Moreover, a weak yet distinct endothermic/exothermic transition was observed immediately after the glass transition, suggesting a melting or crystallization event. This behavior is unexpected for PC, given its typically amorphous nature. To ensure the reliability of the observed thermal transitions, DSC measurements were repeated three independent times under identical experimental conditions. The resulting thermograms showed highly consistent transition temperatures, confirming good reproducibility. On this basis, we confirm that the observed thermal transitions are reproducible. In addition, the sample exhibited a white, opaque appearance, in contrast to the characteristic transparency of neat PC. These findings strongly suggest the formation of a semicrystalline phase during mechanical mixing. Bisphenol-A polycarbonate is generally considered an amorphous polymer; however, literature evidence suggests that under processing conditions, it may exhibit localized ordering or metastable rearrangements within the amorphous phase. As reported by Matsuda et al.,^39^ the formation of metastable phases in bisphenol A polycarbonate can be induced by mechanical stress, leading to enhanced chain mobility at lower temperatures and thereby facilitating local ordering. Furthermore, as reviewed by Di Lorenzo,^40^ mechanical processing may result in partial chain scission and reduced molecular weight, which increases the potential for crystalline packing due to higher chain mobility. In the second cooling cycle of the DSC measurement (Figure 5b), an exothermic crystallization peak with two distinct crystallization temperatures (*T*_c_) was observed at 117.5 and 99.9 °C. The presence of dual *T*_c_ values suggests the formation of polymorphic crystalline structures or domains with differing thermal stability. These findings further support the hypothesis of mechanically induced crystallization during processing. In the present study, this interpretation is further supported by XRD analysis, which indicates that the heat treatment increases the degree of crystallinity and has a strong influence on the development of the structural arrangement. Such structural heterogeneity can arise from thermal history or processing-induced orientation effects. The observed DSC results and XRD peak modifications may indicate partial structural rearrangement or localized ordering within the PC matrix induced by processing and fiber incorporation. The incorporation of SJL fibers into the PC matrix resulted in a systematic decrease in the *T*_g_ with increasing fiber content (5, 10, 15, and 20 wt %), as shown in Table 5 and Figure 6. This decrease in *T*_g_ may be attributed to interfacial effects at the fiber–matrix interface, mainly increased free volume and reduced packing efficiency of polycarbonate chains due to imperfect adhesion. This interpretation is supported by SEM observations, which reveal localized debonding, fiber pull-out, and microvoid formation, particularly at higher fiber loadings. A secondary contribution may arise from the heterogeneous amorphous components of SJL fibers (e.g., lignin), which can locally influence chain mobility.

**Figure 6:**
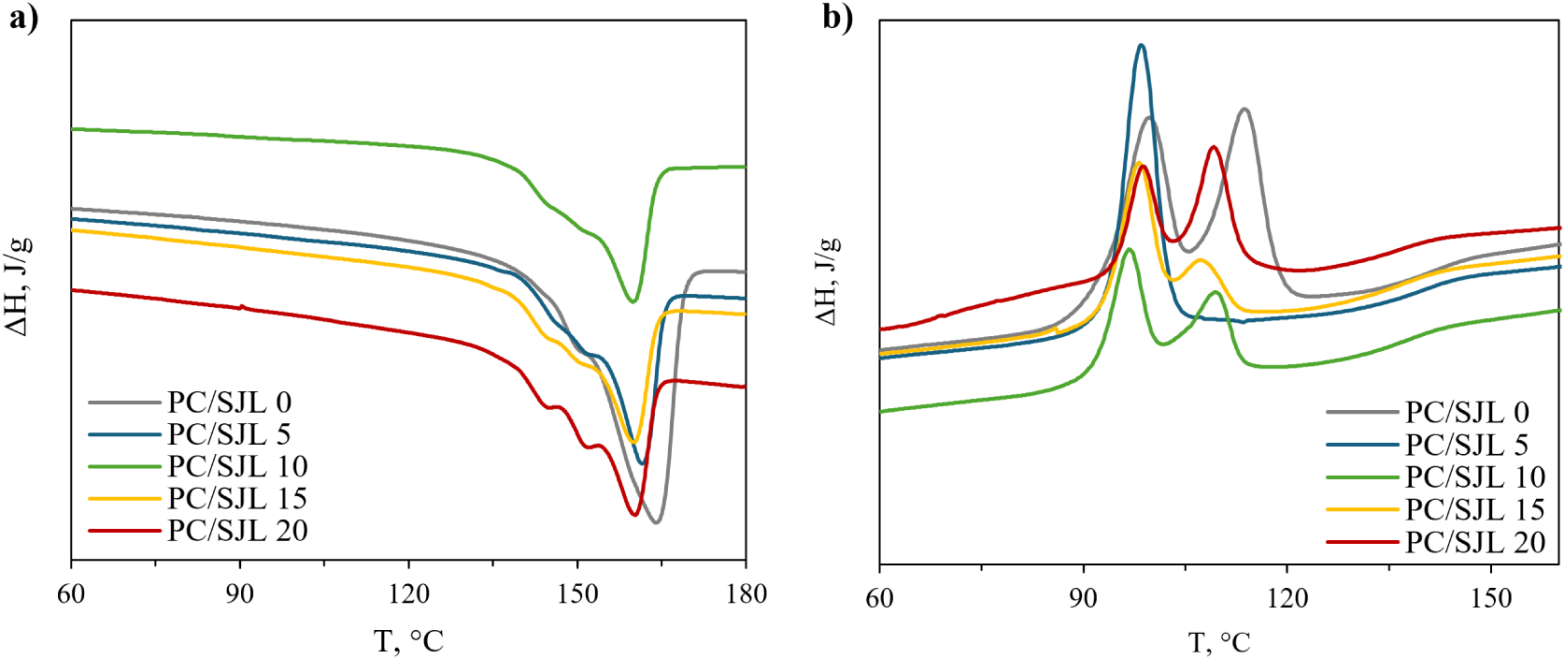
DSC curves of the second heating (a)/cooling (b) cycle of neat PC and PC/SJL composites. Adapted with permission from ref. 23 under the terms of the Creative Commons CC BY license. Copyright 2024 The Authors. Published by TANGER Ltd.

Lignin is an amorphous, hydrophobic aromatic polymer with a Tg around 90–110 °C.^41^ During melt mixing, it is likely that lignin diffuses into the amorphous regions of the PC matrix, enhancing chain mobility and consequently lowering the *T*_g_. Similar reductions in *T*_g_ upon blending with lignin have been reported in biodegradable polyester systems; for instance, blends of poly­(ε-caprolactone) and lignin show *T*_g_ shifts due to partial miscibility and molecular interaction.^42^ The complex chemical composition of SJL fibers comprising cellulose, hemicellulose, and lignin plays a key role in modifying the thermal behavior of the composites. Cellulose is a linear homopolysaccharide consists of repeating cellobiose units, composed of two β-d-glucopyranose subunits linked by β-1,4-glycosidic bonds and typically exhibit a degree of crystallinity ranging from 65% to 70% in natural fibers.^43^ In crystalline regions, cellulose molecules align in parallel, forming microfibrils with unidirectional hydroxyl groups. This crystalline structure provides high tensile strength, stability, and thermal resistance. Hemicelluloses are a heterogeneous group of amorphous polysaccharides present in plant cell walls, characterized by β-(1→4)-linked backbones with an equatorial configuration. This structural arrangement contributes to the flexibility, porosity, and water-holding capacity of the fibers. Major types of hemicelluloses include xyloglucans, xylans, mannans, glucomannans, and β-(1→3,1→4)-glucans.^44^ Composed of various sugar monomers (e.g., xylose, mannose, and arabinose), it has a branched structure. While providing lower mechanical strength, it improves fiber–matrix adhesion and enhances moisture absorption due to its hydrophilic nature. Lignin is a complex aromatic polymer composed of phenylpropanoid units, containing both aromatic and phenolic structures. It functions as a rigidifying agent in the plant cell wall, contributing to the observed changes in the fiber’s thermal behavior.^45^ Overall, the DSC results demonstrate that SJL fibers exhibit good thermal compatibility with the PC matrix and can be incorporated at loadings up to 20 wt % without significantly affecting its characteristic thermal transitions. This suggests their suitability as a thermally stable reinforcing agent.

### FTIR Results

FTIR spectroscopy was employed to evaluate the chemical structure and potential interactions between the PC matrix and SJL fibers in the prepared composites. The FTIR spectrum of neat PC (Figure 7) exhibits characteristic absorption bands corresponding to the functional groups in the PC structure. Absorption bands observed at 2968 cm^–1^, 2920 cm^–1^, 2873 and 2841 cm^–1^ are attributed to C–H stretching vibrations of methyl (−CH_3_) groups, while the absorption band at 1768 cm^–1^ corresponds to C=O stretching of the carboxylic anhydrides.^46^ The absorption band at 1502 cm^–1^ corresponds to C=C stretching vibrations in the aromatic ring, and bands at 1409 cm^–1^ and 1363 cm^–1^ correspond to deformation vibrations of −OH groups. The prominent bands at 1218 cm^–1^, 1185 cm^–1^, 1157 cm^–1^ and 1109 cm^–1^correspond to C–O stretching vibrations, indicative of carbonate and ether linkages within the polymer backbone.^47^ The absorption band at 1012 cm^–1^ corresponds to the stretching vibrations of the C–O–C linkage. Table 6 summarizes the main absorption bands observed in the FTIR spectrum of SJL fibers, which indicate the presence of cellulose, hemicellulose, and lignin through their respective characteristic functional group vibrations. A broad absorption band at 3330 cm^–1^ corresponds to the stretching vibrations of −OH groups in cellulose. The bands at 2915 cm^–1^ and 2899 cm^–1^ are attributed to aliphatic C–H symmetrical stretching in the alkyl chains of cellulose and hemicellulose. The absorption band at 1604 cm^–1^ indicates C=C stretching from the amorphous lignin, while the band at 1100 cm^–1^ is related to the C–O–C glycosidic ether from cellulose. The C–C, C–OH, C–H ring, and side group vibrations of cellulose are observed at 1025 cm^–1^. Furthermore, band at 896 cm^–1^ is related to the COC, CCO, and CCH deformation and stretching in the cellulose.^48^ Figure 7 presents the comparative FTIR spectra of neat PC and PC/SJL composites. A slight shift in the absorption bands at 1109 cm^–1^, 1157 cm^–1^, 1185 cm^–1^, and 1218 cm^–1^ corresponding to C–O functional groups in PC was observed in the composites, which may indicate weak physical interactions such as hydrogen bonding between PC and the hydroxyl groups of the SJL fibers. However, no new absorption bands were detected, and no significant spectral changes were observed with increasing fiber content, suggesting that the incorporation of SJL fibers did not substantially alter the chemical structure of the PC matrix. These results are consistent with previous studies on natural fiber-reinforced polymer composites, in which similar spectral overlaps and slight band shifts have been reported, indicating partial compatibility between the polymer matrix and the lignocellulosic reinforcement.^39^

**Figure 7:**
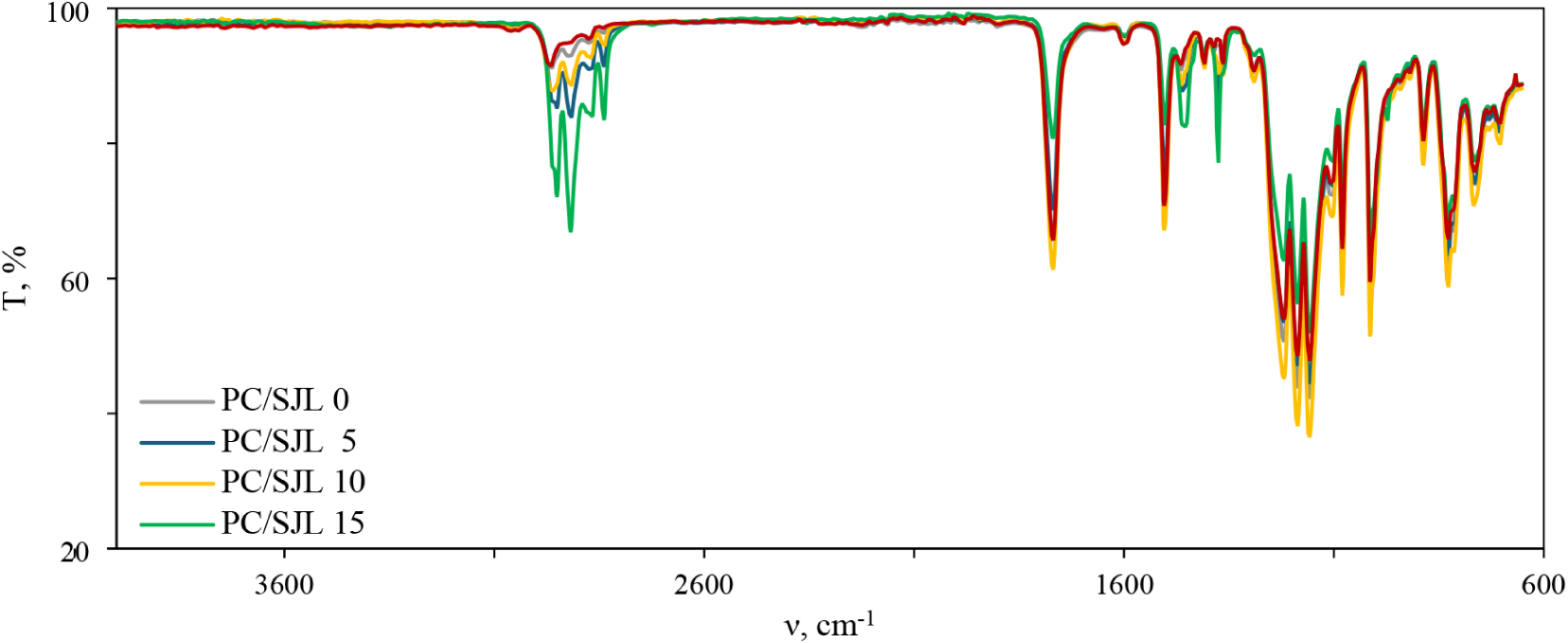
FTIR spectra for neat PC and PC/SJL fiber composites. Adapted with permission ref. 22 under the terms of the Creative Commons CC BY license. Copyright 2025 The Authors. Published by TANGER Ltd.

**Table 6:** FTIR Data Peak Band Assignments for PC and SJL Fiber^a^

	Assignment	Peak position, cm^–1^
**PC**	CH stretching	2968, 2920, 2873, 2841
	C=O stretching	1768
	C=C stretching	1502
	OH deformation	1409, 1363
	C–O deformation	1218, 1185, 1157, 1109
	C–O–C stretching	1012
**SJL**	OH stretching (Cellulose, Hemicellulose)	3334
	C–H symmetrical stretching (Cellulose, Hemicellulose)	2915, 2899
	C=O stretching vibration (Pectin, Waxes)	1730
	C=C stretching (lignin)	1604
	C–O–C glycosidic ether (cellulose)	1100
	C–C, C–OH, C–H ring and side group vibrations (cellulose)	1025
	COC, CCO and CCH deformation and stretching (cellulose)	896

a Data adapted from ref. 22 under the terms of the Creative Commons CC BY license. Copyright 2025 The Authors. Published by TANGER Ltd.

### XRD and XμCT Results

Figure 8 shows the diffraction patterns of SJL fibers and amorphous PC. The diffraction pattern of the SJL fibers indicates that the dominant part consists of cellulose fibers, which form the structurally ordered phase of Cellulose Iβ. Cellulose Iβ crystallizes in a monoclinic crystalline system in space group P21 with unit cell parameters a = 8.2600 Å, b = 10.3880 Å, c = 7.8400 Å, and angles α = γ = 90° and β = 98.8°. Other parts of the SJL sample are structurally ordered phase that belongs to a small quantity of CaCO_3_.

**Figure 8:**
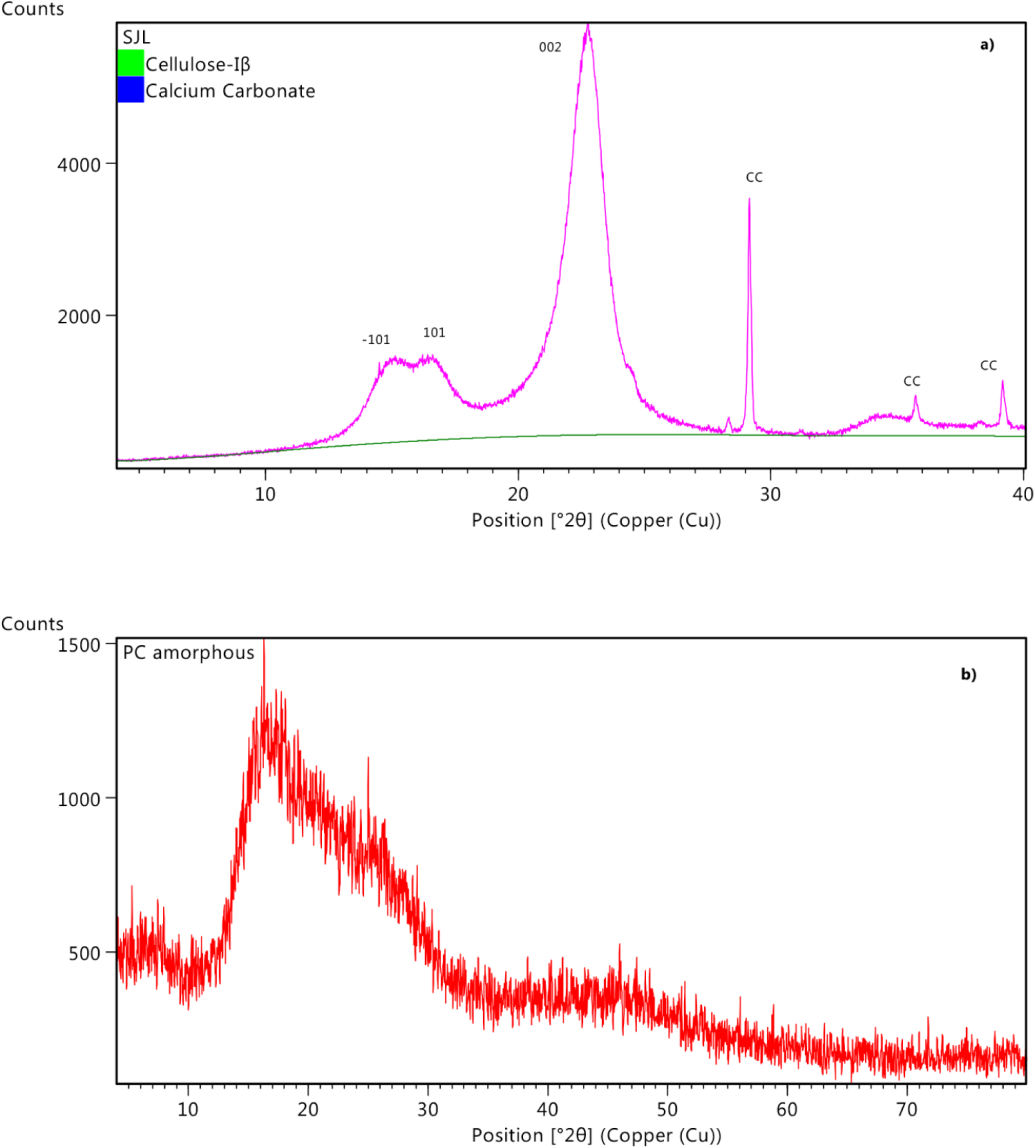
X-ray diffraction pattern of (a) SJL (CC-peaks belong to the CaCO_3_) and (b) PC amorphous.

Figure 9 shows the diffraction patterns of SJL, amorphous PC as well as their composites. The diffraction pattern of PC/SJL 0 belongs to the thermally treated amorphous PC (sample without the addition of SJL), and it shows that thermal treatment influences the increase of structural order in the structure of amorphous PC, indicating that heat treatment increases the degree of structural order and crystallinity of PC and strongly influences the development of its structural arrangement. This structural arrangement is maintained even at 20 wt % SJL. This is a further indication that the addition of SJL fibers does not lead to a complete loss of the structural order of PC as a polymer matrix. At the same time, no cellulose-derived structural order is observed in composites containing SJL in an amount of 5 wt %. In the composite samples with the addition of SJL 10 wt % and more, the occurrence of diffraction maxima originating from structurally ordered cellulose is observed, which is characterized by the strongest diffraction maximum at 22.65° 2 Theta.

**Figure 9:**
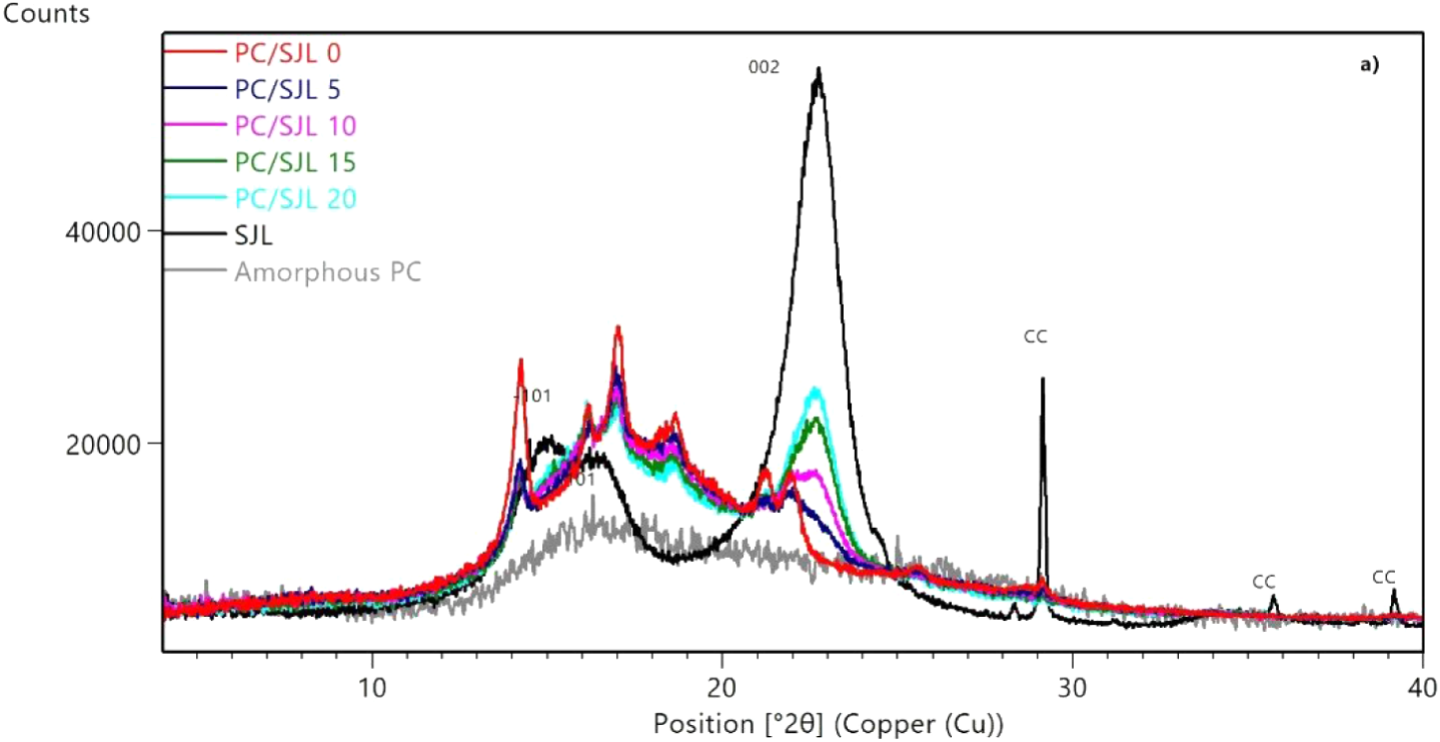
X-ray diffraction patterns of SJL, PC amorphous, and PC/SJL composites.

### Morphology

The distribution of SJL fibers within the polymer matrix, as well as the interactions between the matrix and the fibers, was analyzed using X-ray microcomputed tomography (XμCT) and by using scanning electron microscopy. Figure 10 shows the XμCT reconstruction of the PC/SJL 15 composite. The reconstructed volume of the composite represents a volume of 53.62 mm^3^. From the radiographs, it is visible that the microstructure of the composite consists of voids (closed pores) and inclusions (fibers). The distribution of the voids (air or pores) and inclusions (fibers, SJL) in the PC/SJL composite was determined by VGStudio MAX and module Porosity/Inclusion. From the particle distribution of PC/SJL 15 in the composite, shown at the 3D model (Figure 10), it is visible that the fibers of SJL (as inclusion) were distributed throughout the volume of the sample. The length of the fibers cannot be determined because they are in contact with each other in the volume of the PC polymeric matrix. The volume of the composite, which is occupied by the particles of SPJ fibers, is 10.40 mm^3^ and represents 16.25% of the total volume of the sample. Closed pores present in the microstructure of the composite are irregularly distributed throughout the volume and are not interconnected. The shape of the pores is elliptical, and their sizes range from 0.28 to 0.60 mm. The total volume of the pores is 0.1 mm^3^, which represents 0.01% of the total volume.

**Figure 10:**
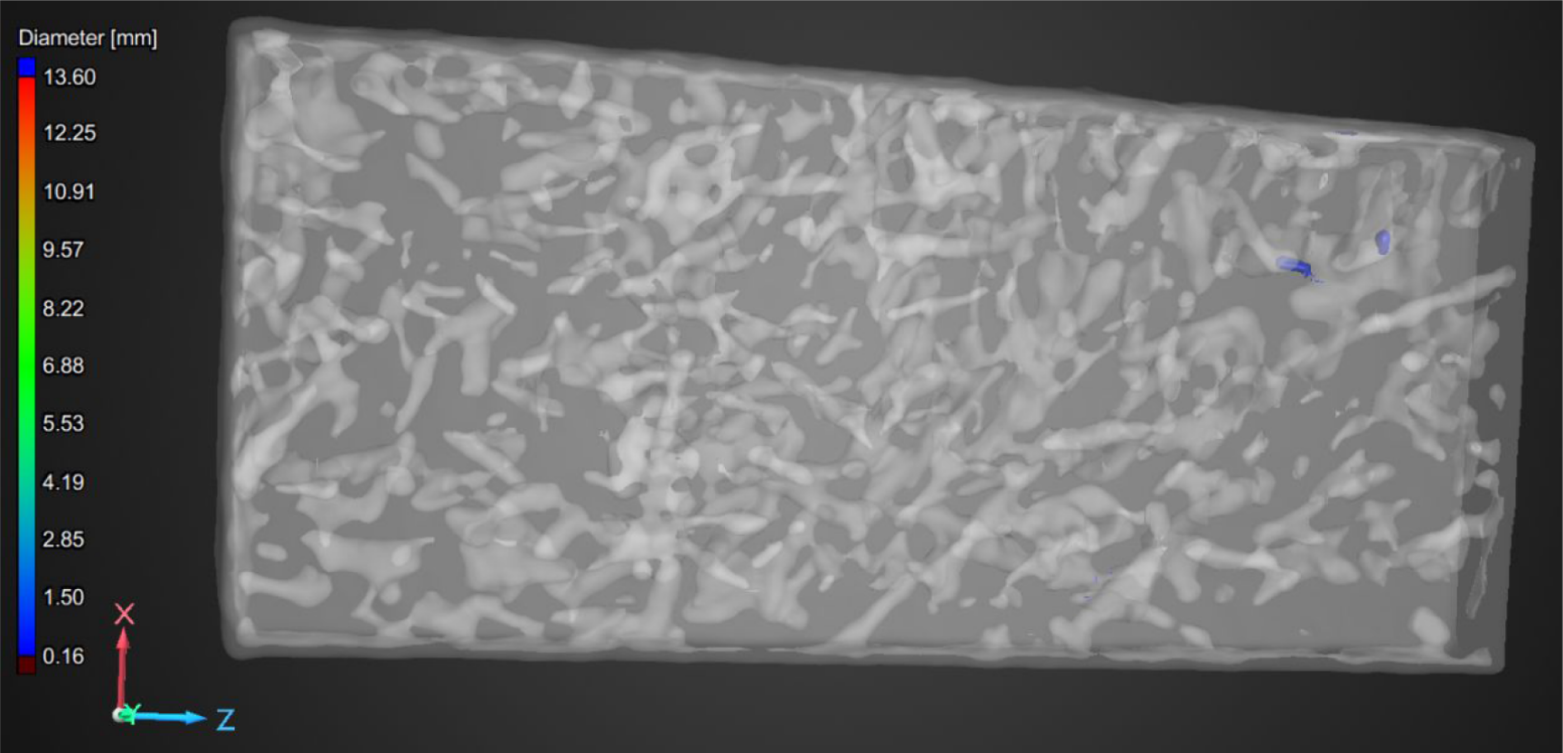
CT reconstruction of PC/SJL 15 composite.

SEM images were taken on the fracture surfaces of the composites after mechanical testing, capturing the morphology of the failure surface and providing insight into the failure mechanism. Figure 11 displays the SEM micrographs of neat PC and PC reinforced with various contents of SJL fibers. SJL fibers (Figure 11f) measure 10–100 μm in width. Surface analysis reveals wrinkles, grooves, and kinks that could facilitate enhanced mechanical interlocking between the fibers and the PC matrix. The surface morphology of neat PC (Figure 11a) reveals a rough and irregular texture with visible microvoids, which are characteristic features of brittle fracture surfaces in unreinforced thermoplastics.^49^ Such microvoids, often associated with craze formation, have been frequently observed in SEM studies of polycarbonate fracture surfaces under tensile or impact loading conditions As the SJL fiber content increases from 5 to 20 wt %, progressive changes in composite microstructure are evident. SEM micrographs (Figure 11b–d) show relatively homogeneous dispersion of SJL fibers within the PC matrix, particularly at lower fiber content. The fibers appear intertwined and embedded, forming a continuous reinforcing network, indicating good interfacial adhesion and mechanical interlocking between fibers and the matrix. This morphology supports efficient stress transfer from the matrix to the fibers, consistent with observed increases in tensile strength at intermediate fiber contents. The improved fiber–matrix bonding observed in composites with lower fiber content is consistent with literature findings. George et al.^50^ and Herrera-Franco and Valadez-Gonzalez^51^ reported that optimal fiber loading promotes better wetting and adhesion, resulting in enhanced mechanical performance of natural fiber-reinforced polymer composites. Excessive fiber content often leads to the formation of voids and reduced mechanical performance because of fiber agglomeration. These morphological observations obtained by SEM complement the previously discussed thermal and mechanical characterizations. Relatively uniform dispersion and good interfacial adhesion of SJL fibers, particularly at 10 and 15 wt %, correlate with the observed improvements in tensile strength (Table 3) and stiffness (Table 2), as confirmed by the DMA results. Furthermore, efficient fiber–matrix bonding may contribute to the moderate reduction in thermal stability observed in TGA, since the incorporation of lignocellulosic fibers introduces thermally less stable domains within the composite. The slight downward shift in *T*_g_ values observed in both DSC and DMA analyses can be attributed to partial interpenetration of lignin-rich fiber surfaces into the amorphous regions of the PC matrix, as supported by the fiber–matrix entanglement evident in the SEM micrographs. This combined evidence supports the hypothesis that mechanical interlocking and interfacial compatibility between SJL fibers and the PC matrix play a critical role in determining the overall performance of the composites. The generally weak interfacial interactions observed across the investigated PC/SJL composites can be attributed to the inherent incompatibility between the hydrophilic lignocellulosic nature of untreated SJL fibers and the hydrophobic PC matrix, resulting in limited interfacial adhesion and inefficient stress transfer across the fiber–matrix interface. This behavior is widely reported in natural fiber–reinforced composites and is primarily associated with differences in polarity and surface energy, which hinder effective interfacial bonding. Improved mechanical performance observed at 10–15 wt % is primarily attributed to optimized physical reinforcement and favorable morphological features rather than strong chemical adhesion. In particular, the comparatively superior behavior at 15 wt % is likely related to a more uniform fiber dispersion, a suitable fiber aspect ratio distribution, reduced void formation, and enhanced stress transfer through mechanical interlocking between the fibers and the matrix. In contrast, the microstructure of the PC/SJL 20 composite (Figure 11e) reveals several morphological defects, including increased porosity, fiber pull-out, and fiber agglomeration. These features are indicative of poor fiber dispersion and weakened interfacial bonding. Such deterioration in fiber–matrix interaction at higher fiber loading correlates well with the mechanical results, which show a reduction in tensile strength and elongation at break for the PC/SJL 20 sample.^22^ The presence of interfacial gaps and loosely bound fibers limits efficient stress transfer, thereby promoting early failure under load. SEM observations, together with the mechanical behavior at higher untreated SJL fiber content, indicate that interfacial bonding is locally limited. This is consistent with the hydrophilic nature of SJL fibers and the hydrophobic character of polycarbonate. The observed limited interfacial adhesion indicates that SJL fiber surface modification, for example, via alkali treatment or silane coupling, represents a promising strategy to improve fiber–matrix compatibility and consequently enhance the mechanical performance of the PC/SJL composites. The interfacial adhesion can be significantly improved through alkali treatment (e.g., NaOH), which removes surface impurities such as waxes, hemicellulose, and lignin while increasing surface roughness, thereby enhancing mechanical interlocking and the effective interfacial contact area. Silane coupling agents further promote interfacial bonding by improving the chemical compatibility between hydroxyl groups on the fiber surface and the polymer matrix, resulting in more efficient stress transfer. In addition, compatibilizers or surface functionalization strategies can enhance interfacial performance through chemical interactions and improved wetting of the fiber surface by the matrix, ultimately leading to better load transfer efficiency.

**Figure 11:**
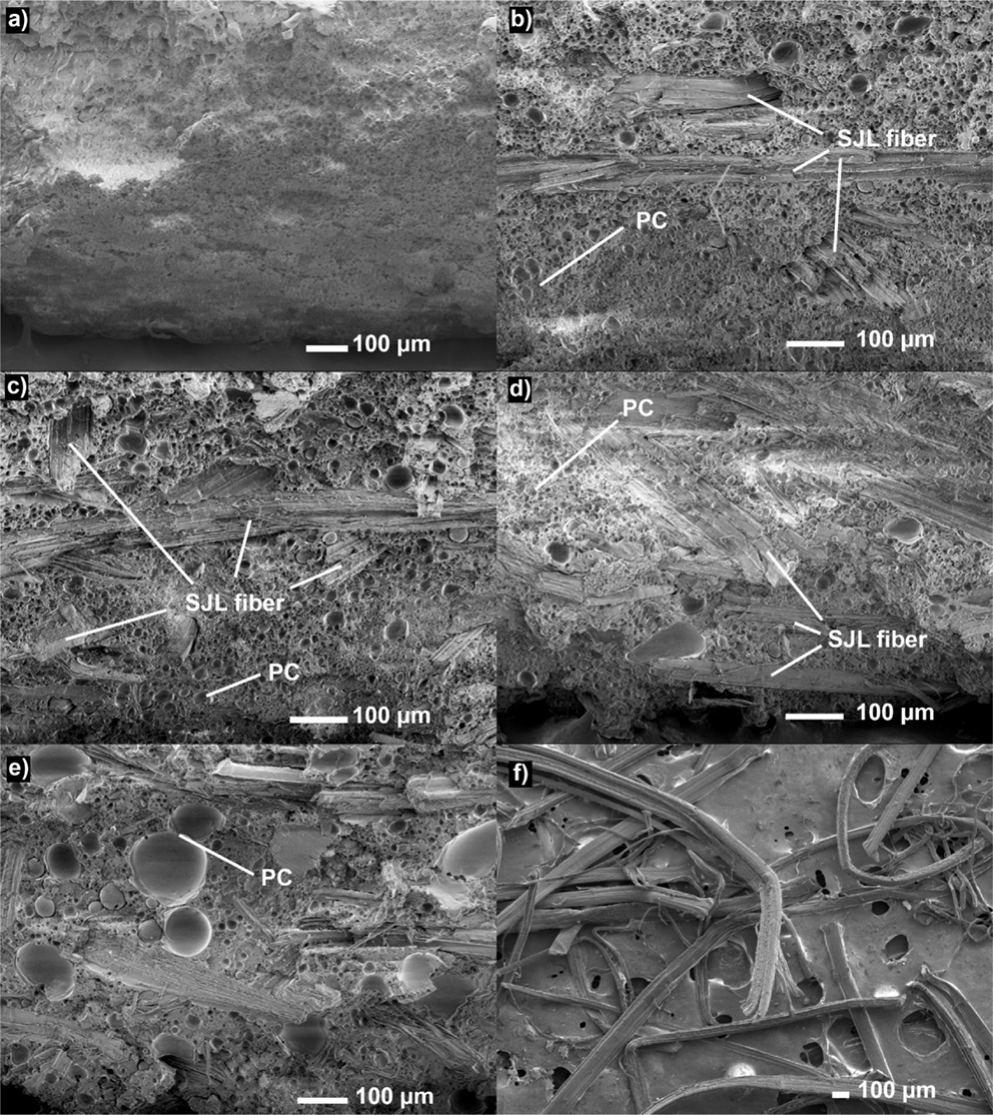
SEM images of the fracture of surface of a) PC/SJL 0; b) PC/SJL 5; c) PC/SJL 10; d) PC/SJL 15, e) PC/SJL 20 composites, and f) SJL fibers. Adapted with permission from ref. 22 under the terms of the Creative Commons CC BY license. Copyright 2025 The Authors. Published by TANGER Ltd.

### TGA Results

Thermal stability is a critical parameter for evaluating the suitability of polymer composites in high-temperature applications. In this study, thermogravimetric analysis was employed to investigate the thermal degradation behavior of neat PC and PC/SJL composites. Thermogravimetric (TG) and derivative thermogravimetric (DTG) curves were analyzed to determine key thermal parameters, including the onset degradation temperature (T_5%_), the temperature at the maximum decomposition rate (*T*_max_), and the residual mass (R). These parameters provide insight into the influence of SJL fiber incorporation on the thermal stability of the composites. The resulting TG curves are presented in Figure 12a, while the corresponding DTG curves are shown in Figure 12b. Characteristic values obtained from the TG and DTG curves are summarized in Table 7.^58^ Lignocellulosic fibers are primarily composed of hemicellulose (20–40%), cellulose (40–60%), and lignin (10–25%), each featuring a complex molecular structure. These components are thermally unstable and begin to degrade at relatively low temperatures. In general, the thermal degradation of natural fibers proceeds in multiple stages across distinct temperature ranges.^52^ The first stage is typically associated with the degradation of pectin, hemicellulose, and parts of lignin, while the second stage corresponds to the decomposition of α-cellulose. In this study, the TG curve of SJL fibers under a nitrogen atmosphere revealed two main stages of thermal decomposition. The DTG curve (Figure 12b) displays three degradation peaks at 167.9 °C, 378.7 °C, and 460.2 °C, indicating a three-step thermal degradation process. The temperature corresponding to a 5% weight loss (T_5%_) marks the onset of degradation for both neat PC and PC/SJL composites, as presented in Table 7. A minor mass loss of 3.89% observed below 200 °C is attributed to the evaporation of adsorbed moisture.^52^ The second, major mass loss at 378.7 °C, accounting for 73.82% of the total weight, is associated with the degradation of hemicellulose, cellulose, and lignin. Complete degradation of lignin occurs at higher temperatures (around 550 °C), while the residual mass of 21.10% at 600 °C is primarily attributed to the presence of nonvolatile solid constituents in the SJL fibers.

**Figure 12:**
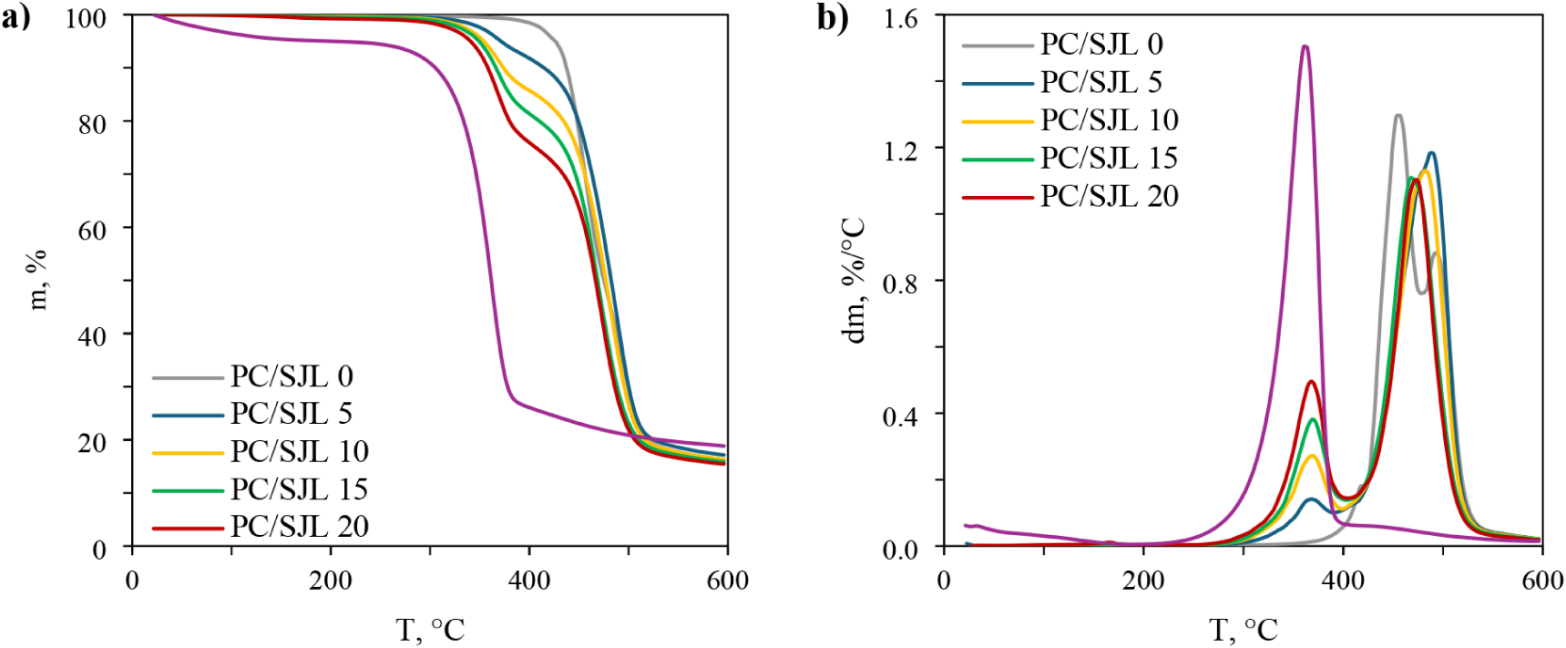
(a) TG and (b) DTG curves of neat PC, SJL fibers, and PC/SJL composites. Adapted with permission from ref. 23 under the terms of the Creative Commons CC BY license. Copyright 2024 The Authors. Published by TANGER Ltd.

**Table 7:** TGA Results of Neat PC, SJL Fibers, and PC/SJL Composites^a^

	T_max_, **°**C						Δm, %		
Sample	1	2	3	R, %	T_5%_, **°**C	**T**_end_, **°**C	1	2	3
PC/SJL 0	-	-	493.8	16.27	426.2	512.9	0.01	4.24	48.33
PC/SJL 5	161.6	369.5	460.0	17.18	371.5	509.1	0.19	7.04	75.52
PC/SJL 10	164.2	368.9	489.6	16.36	354.3	504.9	0.33	13.67	69.58
PC/SJL 15	166.1	370.1	483.7	15.96	349.4	498.3	0.47	18.54	64.96
PC/SJL 20	166.0	368.8	468.7	15.56	342.9	497.5	0.72	23.78	59.71
SJL	167.9	378.7	460.2	21,10	275.4	460.5	3.89	73.82	1.20

a Data adapted from ref. 23 under the terms of the Creative Commons CC BY license. Copyright 2024 The Authors. Published by TANGER Ltd.

The neat PC undergoes a single-step thermal degradation process and remains thermally stable up to 426 °C. Decomposition proceeds up to 512.9 °C, marking the completion of thermal degradation (T_end_). Montaudo et al. proposed a thermal degradation mechanism of PC.^53^ In the initial stage, an intramolecular exchange reaction occurs within the polymer backbone, leading to the formation of cyclic carbonate structures. This process is a characteristic feature of PC degradation at elevated temperatures. The formation of such structures reduces chain integrity and initiates the breakdown of the polymer matrix. In the second stage, thermal cleavage of the carbonate bond takes place, resulting in the elimination of carbon dioxide (CO_2_) through a 1,3-rearrangement mechanism. This reaction leads to the formation of ether linkages between bisphenol A units, altering the polymer’s chemical architecture and accelerating the degradation process. The evolution of CO_2_, commonly observed during thermogravimetric analysis, is indicative of scission of the carbonate groups. Finally, in third stage, hydrolytic degradation of the carbonate linkage occurs in the presence of water, producing phenolic end groups along with additional CO_2_ release. This reaction becomes particularly relevant under humid conditions or during prolonged exposure to elevated temperatures, contributing to increased mass loss and polymer discoloration. In contrast, PC/SJL composites exhibit a three-step thermal degradation. The first two stages are attributed to the thermal decomposition of the lignocellulosic fiber components, while the third stage corresponds to the degradation of the PC. The initial degradation temperature (T_5%_) of PC/SJL composites is lower than that of neat PC due to the intrinsically lower thermal stability of SJL fibers. Notably, the composite with the highest fiber loading (20 wt %) exhibits the lowest T_5%_ value (275.4 °C), indicating that fiber content strongly influences the onset of thermal degradation. These results are consistent with previous studies on polymer composites reinforced with natural fibers. For example, M. N. Belgacem and A. Gandini reported that, although natural fibers can provide some degree of thermal insulation, their relatively low thermal degradation onset typically between 200 and 300 °C often results in a decrease in the initial decomposition temperature of fiber-reinforced composites.^54^

### Swelling Results

Figure 13 represents the percentage of absorbed moisture as a function of time for neat PC and PC/SJL composites following their immersion in distilled water. The water absorption curve of each sample initially exhibits a linear path and gradually reaches equilibrium, where the behavior of the sample conforms to the Fickian diffusion process. The neat PC exhibited lower swelling compared to the PC/SJL composites, and this is because the amount of matrix is higher at the lower fiber weight percentage. The matrix material is hydrophobic, which restricts the interaction of the water molecules with the fiber content.^55–57^ Higher fiber content increased water absorption due to increased contact between water molecules and fibers. Natural fibers are hydrophilic due to the presence of cellulose, hemicellulose, and lignin, which contain hydroxyl groups.^58^ When such fibers are added to a hydrophobic polymer matrix like polycarbonate, they create pathways through which water penetrates the material. The process occurs through three main mechanisms: diffusion through microcracks within the polymer matrix; capillary transport through microscopic channels at the interface between the fibers and the matrix; and interfacial accumulation in voids and defects at the fiber–polymer junction, where it binds to fibers via hydrogen bonds.^56^ A slight decrease in swelling values was observed with increasing SJL fiber content after approximately 150 h of immersion. This trend indicates a saturation point in water absorption capacity, which is beneficial, as excessive swelling could compromise the mechanical integrity of the composites. Similar behavior has been reported in other studies on natural fiber-reinforced composites, where water uptake decreased beyond a certain fiber content.^58^

**Figure 13:**
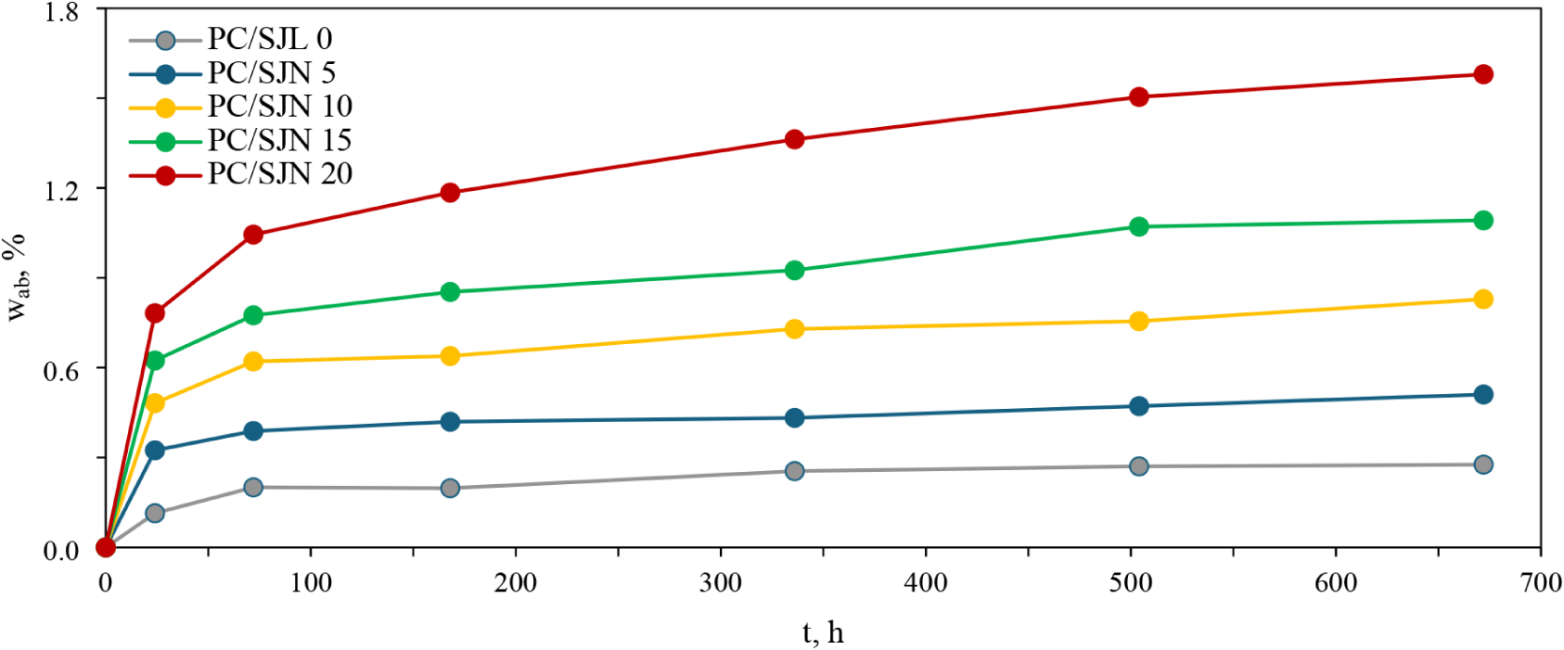
Water absorption of PC/SJL composites based on the exposure time.

## Conclusions

Compared to commonly used natural fibers and agro-waste-derived fillers such as flax, kenaf, or jute fibers in polymer composites, *Spartium junceum* *L.* (SJL) fibers remain largely unexplored as a reinforcing phase in thermoplastic matrices. Similar trends in stiffness enhancement and ductility reduction have been reported for biocomposites such as walnut shell/pine needle ash-reinforced polylactic acid systems and *Pinus roxburghii* fiber-based composites. This work presents, for the first time, a systematic structure–property investigation of polycarbonate/*Spartium junceum* L. fiber composites, establishing correlations between fiber content, interfacial morphology, thermal relaxation, and mechanical performance. The novelty of this work resides in introducing SJL fibers as a sustainable reinforcement for advanced polymer composite applications. In addition, it highlights the governing role of the interfacial structure—particularly free volume, dispersion state, and fiber–matrix interactions—in determining composite performance. The incorporation of SJL fibers modifies the segmental dynamics of the PC matrix, as reflected by changes in *T*_g_. The observed decrease in *T*_g_ with increasing fiber content may be attributed to increased interfacial free volume arising from imperfect fiber–matrix adhesion. This interpretation is supported by SEM observations, which show localized debonding, fiber pull-out, and microvoid formation, particularly at higher fiber loadings. These features indicate reduced interfacial continuity and enhanced local chain mobility in the amorphous regions of the matrix. Thermal analysis of PC and PC/SJL composites reveals that fiber reinforcement not only alters the *T*_g_ but may also induce semicrystalline ordering in the otherwise amorphous PC matrix, particularly under mechanical shear. These findings corroborate earlier observations of mechanically induced crystallization in thermoplastics and highlight the structural complexity introduced by natural fiber additives. The incorporation of SJL fibers enhances tensile strength and stiffness, especially at lower fiber contents, but reduces elongation at break. These results demonstrate the potential of SJL fibers as sustainable reinforcement for polymer composites. XRD analysis showed that neat SJL fibers crystallize in the Cellulose Iβ form, while neat PC, although amorphous, exhibits partial structural ordering enhanced by heat treatment. This ordering is preserved up to 5 wt % SJL, but higher fiber contents disturb the structural arrangement of PC, as indicated by the loss of its dominant diffraction peak. Nevertheless, PC-derived diffraction maxima remain, confirming that complete structural disorder does not occur. At 20 wt % SJL, characteristic cellulose peaks appear, revealing the contribution of crystalline cellulose to the composite structure. SEM analysis supports the conclusion that an intermediate SJL fiber content (10–15 wt %) achieves optimal dispersion and interfacial bonding, which are essential for reinforcing the PC matrix without compromising its structural integrity. The incorporation of SJL fibers into the PC matrix alters the thermal degradation of the composites. While neat PC undergoes single-step degradation with high thermal stability, lignocellulosic fibers introduce earlier degradation stages, reflecting their thermal sensitivity. Despite this, the residual mass at elevated temperatures indicates the formation of a more thermally stable char structure. These findings emphasize the need to optimize fiber content and processing conditions to balance mechanical reinforcement and thermal performance in composites. PC/SJL composites exhibit increased swelling due to the hydrophilic nature of SJL fibers, but a saturation point at higher fiber contents improves dimensional stability. The research conducted in this work aims to develop sustainable natural fiber-reinforced composites with potential engineering applications, including possible use in the automotive industry. The PC/SJL composites investigated in this work may be particularly interesting for interior automotive components; however, the studies performed represent an initial assessment rather than full industrial validation. Therefore, additional testing related to specific automotive industry requirements, such as impact resistance, flame retardancy, and UV stability, is necessary for further investigations.

## Abbreviations

PC: Polycarbonate
SJL: *Spartium junceum* L.
XμCT: X-ray microcomputed tomography
